# Impact of the Coronavirus Disease 2019 Pandemic on Medical Practices in Awaji Island

**DOI:** 10.31662/jmaj.2023-0107

**Published:** 2023-11-16

**Authors:** Yoshihiko Omori, Naoto Oka, Yasukuni Suzuki, Masayuki Shima, Hiroki Nishikawa, Kenzo Tsuzuki

**Affiliations:** 1 Department of Otorhinolaryngology, Hyogo Prefectural Awaji Medical Center, Sumoto, Japan; 2 Department of Surgery, Hyogo Prefectural Awaji Medical Center, Sumoto, Japan; 3 Department of Public Health, Hyogo Medical University, Nishinomiya, Japan; 4 Department of 2nd Internal Medicine, Osaka Medical and Pharmaceutical University, Takatsuki, Japan; 5 Department of Otorhinolaryngology-Head and Neck Surgery, Hyogo Medical University, Nishinomiya, Japan

**Keywords:** COVID-19, pandemic, infection rate, restrictions, working hours, epidemiological survey

## Abstract

**Introduction:**

Since the first confirmed case of coronavirus disease 2019 (COVID-19) in China, COVID-19 continues to be a global threat and exerts a significant impact on medical practices. This study aims to investigate the impact of the COVID-19 pandemic on medical practices in Awaji Island, a remote island in Japan.

**Methods:**

First, we conducted a survey on the epidemiological characteristics of COVID-19 on Awaji Island before and during the pandemic. Next, using a questionnaire, we conducted a survey with doctors working full time at Hyogo Prefectural Awaji Medical Center, which is the only designated infectious disease hospital on Awaji Island.

**Results:**

The COVID-19 infection rate of Awaji Island was lower than that of Hyogo Prefecture and of Japan as a whole, although the peaks occurred simultaneously. Outpatient visits as well as hospitalized patients, i.e., inpatients, decreased during the pandemic as a result of restrictions on surgeries and hospitalizations, with no changes in the disease composition ratio. The results of the questionnaire show that during the pandemic, doctors working full time at our hospital worked less and slept more. Furthermore, data obtained from the Medical Affairs Department showed a decrease in overtime hours worked and an increase in the number of days of paid holidays taken.

**Conclusions:**

Epidemiologically, the impact of the COVID-19 pandemic on Awaji Island showed a similar trend to that in Japan, but the results of the survey questionnaire indicated that doctors working full time at our hospital were not necessarily adversely affected.

## Introduction

Since the world’s first case of coronavirus disease 2019 (COVID-19), which is caused by infection with the novel severe acute respiratory syndrome coronavirus 2 (SARS-CoV-2), was confirmed in Wuhan, China in December 2019 ^(1), (2)^, COVID-19 has continued to pose as a global threat. In January 2020, within the framework of suspected disease surveillance, a system was put in place in Japan to detect patients with COVID-19, for which multiple epidemics involving various mutations of SARS-CoV-2, including the Alpha (BA.1), Delta (BA.2), and Omicron (BA.5) strains, have occurred ^(3)^.

COVID-19 continues to pose a threat to health care, thereby forcing healthcare workers to take ongoing measures against infection. In Japan and other countries around the world, the COVID-19 pandemic has affected health care systems and disrupted economic activities in various ways. There was a time in the medical field when a shortage of hospital beds due to the hospitalization of patients with COVID-19 became a problem. Accordingly, it is important to implement policies that adequately factor in the occupational dimension of risk ^(4)^.

**Figure 1.**
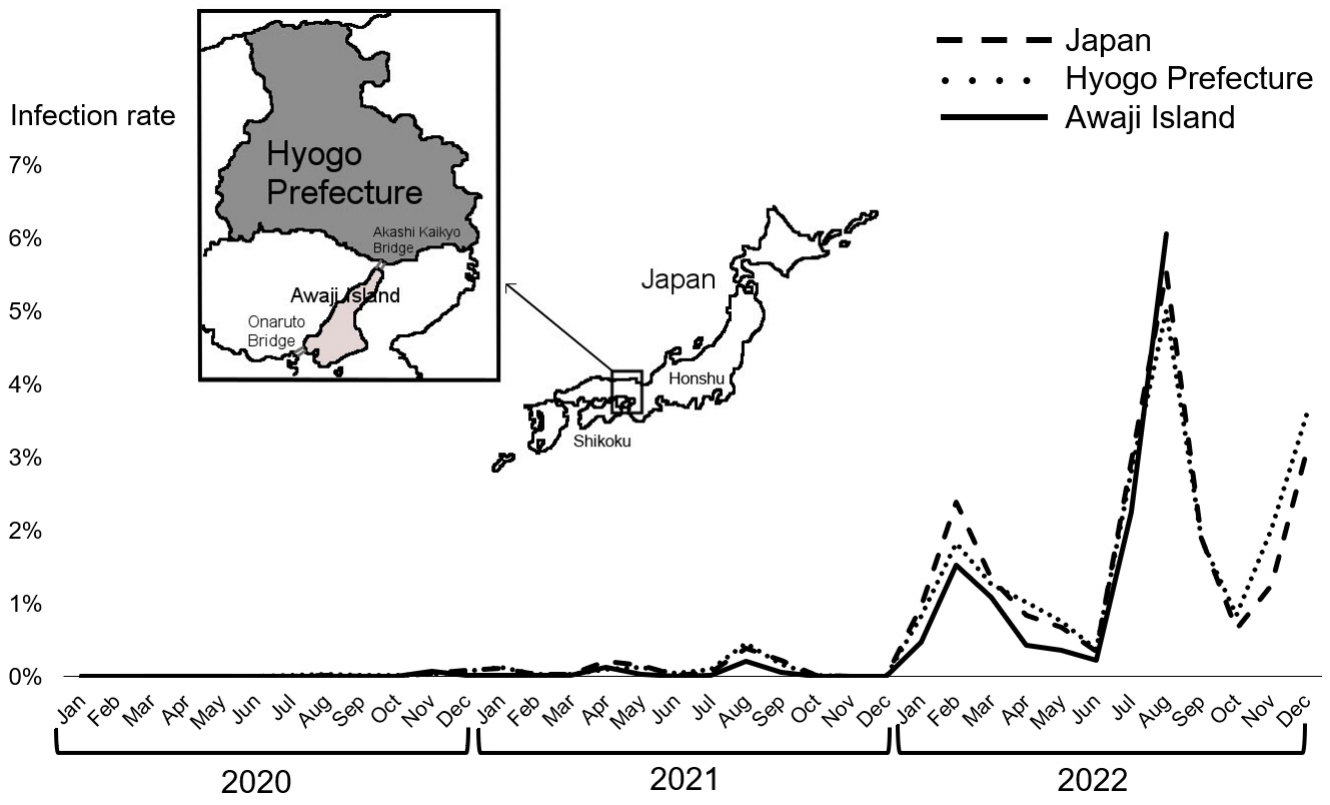
Time course of the COVID-19 infection rates and geographical relationship among Honshu, Awaji Island, and Shikoku. Japan (fine dotted line), Hyogo Prefecture (coarse dotted line), and Awaji Island (solid line).

Awaji Island is the largest island in the Seto Inland Sea and the 11th largest in Japan (Figure 1). Awaji Island has a high aging population of approximately 130,000, with 38% being age 65 years or older and 20% being 75 years or older, as of February 2023 ^(5)^. Geographically, Awaji Island does not have a land connection to the mainland islands of Honshu and Shikoku. However, the Kobe-Awaji-Naruto Expressway connects Awaji Island to Honshu by the Akashi Kaikyo Bridge (the northern part) and Shikoku by the Onaruto Bridge (the southern part). These toll bridges are the only land routes to Awaji Island. No railways operate on Awaji Island, and transportation within the island is limited to private cars and local buses.

According to the Hyogo Prefecture Medical Institution Information System, as of August 2023, Awaji Island has 11 general hospitals, 99 private clinics, and 72 dental clinics. Hyogo Prefectural Awaji Medical Center (hereinafter referred to as “our hospital”) is the only hospital on Awaji Island designated for treating infectious diseases ^(6)^, and as such, the COVID-19 pandemic has led to necessary changes in the medical system. At the time of submission of this paper, our hospital is the only facility on Awaji Island that can treat COVID-19 infection. A COVID-19 countermeasure meeting was held at least 2-3 times a week, and measures were taken to prevent the spread of COVID-19 in hospitals and maintain the capacity to treat urgent cases other than COVID-19. In our hospital, patients infected with COVID-19 who required hospitalization were admitted to a dedicated COVID-19 ward. However, the number of beds in the COVID-19 ward was limited, and some patients with COVID-19 could not be accepted at our hospital. Furthermore, during the peak of the pandemic, we were forced to convert temporarily the general wards on the same floor or the intensive care unit (ICU) as COVID-19-dedicated wards to accept patients with COVID-19. Under this situation, there were a series of cases where our hospital could not provide the same medical care as before the pandemic. For example, surgical cases requiring ICU admission had to be referred to other hospitals, and less urgent operations had to be postponed.

In the future, there is a possibility that emerging infectious diseases other than COVID-19 will spread, and it is conceivable that these will have the same impact on medical practices in hospitals. The present study aims to evaluate how the measures taken by our hospital during the pandemic have affected medical practice and to discuss how physicians should work under the influence of emerging infectious diseases.

## Materials and Methods

The study period was from January 2016 to December 2022. In this study, “during the pandemic” is defined as the period after January 2020 when the first patient with COVID-19 was confirmed in Japan, and the “pre-pandemic” period was the period prior to January 2020, i.e., from January 2016 to December 2019.

This study (i) analyzed data on people infected with COVID-19 in Japan as a whole, in Hyogo Prefecture, and on Awaji Island, as well as patients treated at Hyogo Prefectural Awaji Medical Center for the epidemiological survey and (ii) surveyed doctors who were working full time at our hospital as of April 1, 2022.

This study was approved by the Hyogo Prefectural Awaji Medical Center Ethics Committee (Approval No.: 22-1). This study was performed in accordance with the principles of the Declaration of Helsinki.

### Epidemiological study on the impact of the COVID-19 pandemic on Awaji island

As the regional characteristics of Awaji Island emerged, a comparative epidemiological study on trends in the number of people with COVID-19 in (i) Japan, (ii) Hyogo Prefecture, and (iii) Awaji Island during the pandemic was conducted. Data on the reported number of patients with COVID-19 were obtained from (i) the Ministry of Health, Labour and Welfare of Japan ^(7)^, and (ii, iii) Hyogo Prefecture ^(8)^. Infection rates were shown as percentages of the population. As the September 2022 survey on the number of patients with COVID-19 was revised and public health centers no longer collect data on the number of patients, data on patients after September 2022 could not be obtained. To evaluate the changes in the population influx to Awaji Island before and after the pandemic and the number of infected people on the island, we also investigated the traffic volume on the Kobe-Awaji-Naruto Expressway and the Akashi Kaikyo Bridge ^(9)^.

### Epidemiological study on the influence of the COVID-19 pandemic on Hyogo prefectural Awaji medical center

We investigated the number of ordinary and emergency outpatients and inpatients in our hospital in the pre- and during pandemic periods by year. We focused on inpatients, the number of patients with COVID-19 per month, severely ill patients with COVID-19, and patients without COVID-19 in our hospital during the pandemic. Severely ill patients with COVID-19 were defined as patients on ventilators or extracorporeal membrane oxygenation in ICUs according to the Ministry of Health, Labour and Welfare of Japan. Furthermore, we investigated the relationship between changes in the number of inpatients and the restrictions on medical treatment at our hospital. The restrictions on medical treatment were confirmed from the records of countermeasures against COVID-19. Countermeasure meetings were held 2-3 times a week between May 2020 and April 2023. The minutes of these meetings were provided by the General Affairs Division of our hospital.

Furthermore, the disease composition of inpatients for every year was examined according to data obtained from the Medical Affairs Department of our hospital. The disease composition was classified as malignant neoplasm, otorhinolaryngology disease, respiratory disease, cardiovascular disease, gastrointestinal disease, musculoskeletal disease, neurological disease, trauma, and others, according to the clinical department to which patients were admitted.

### Influence of the COVID-19 pandemic on doctors working full time at Hyogo prefectural Awaji medical center

We investigated how the COVID-19 pandemic changed the overtime hours and number of paid holidays taken by all doctors working full time at our hospital and conducted a comparison between the pre-pandemic period and the period during the pandemic. Data provided by the General Affairs Division of our hospital were analyzed and represented as mean values by year. However, data from 2016 could not be obtained by the General Affairs Division. The number of days of missed work by full-time doctors because of infection with COVID-19 was excluded from the analysis.

To evaluate the influence of the COVID-19 pandemic on the work status of doctors working full time at our hospital during the pandemic, we conducted a survey using a questionnaire composed of the following four items on physical and psychological changes: (a) change in working hours (increased, no change, or decreased); (b) change in sleep time (increased, no change, or decreased); (c) patient hospitalization postponed (postponed or not postponed); and (d) consultation about COVID-19 from a patient (yes or no). The questions were determined in light of the recent work environment for doctors working in community-based general hospitals.

Doctors who were working full time at our hospital as of April 1, 2022 were enrolled in this study. Residents, part-time doctors, nurses, and clinical laboratory technologists and technicians were excluded. Before the survey, Informed consent was obtained in person to obtain information on the participants’ gender, clinical department, and years of experience as a doctor. The questionnaires were printed on paper before being distributed to the participants. The survey period was from April 14, 2022 to August 15, 2022.

## Results

### Characteristics of patients infected with COVID-19 on Awaji island

Epidemiologically, the trend of the COVID-19 infection rate on Awaji Island was parallel to that in Hyogo Prefecture and in Japan as a whole (Figure 1). The timing of the peak infection rate was consistent in all three regions, as shown in August 2021 and February and August 2022. The COVID-19 positivity rate on Awaji Island was lower than that in Hyogo Prefecture and in Japan as a whole. In August 2022, the COVID-19 infection rate on Awaji Island exceeded those in Hyogo Prefecture and Japan as a whole for the first time. This coincided with an increase in traffic on the Akashi Kaikyo Bridge and Onaruto Bridge ^(9)^.

### Changes in the number of outpatients at Hyogo prefectural Awaji medical center

**Figure 2.**
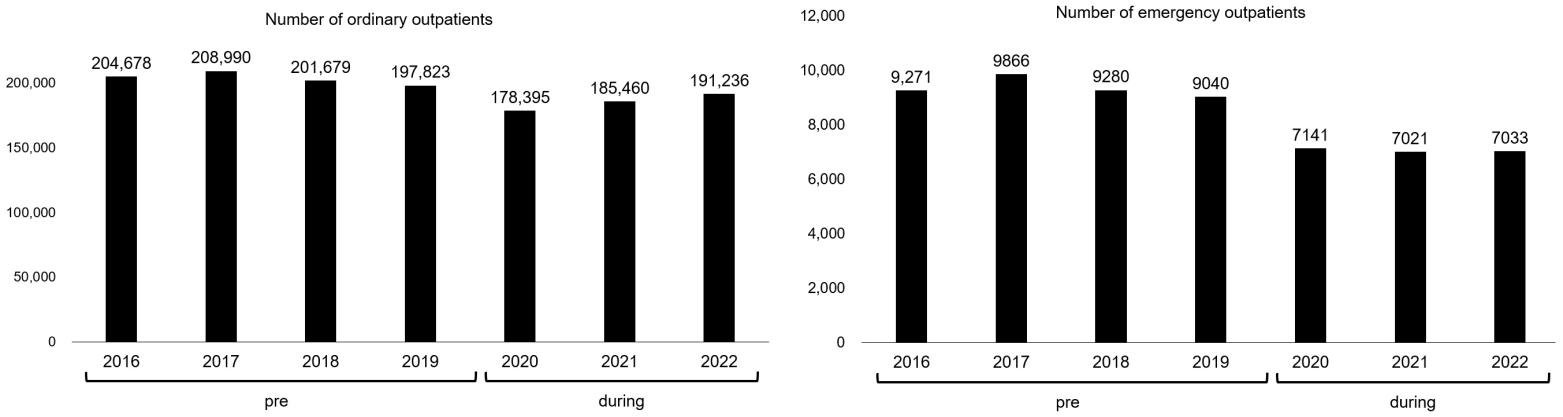
Changes in the number of outpatients. Annual number of ordinary and emergency outpatients at our hospital in the pre- and during the pandemic periods.

Figure 2 shows the comparisons between the number of ordinary and emergency outpatients in the pre- and during the pandemic periods at our hospital. During the pandemic, there was a downward trend in the number of ordinary and emergency outpatients at our hospital.

### Changes in the number of inpatients at Hyogo prefectural Awaji medical center

**Figure 3.**
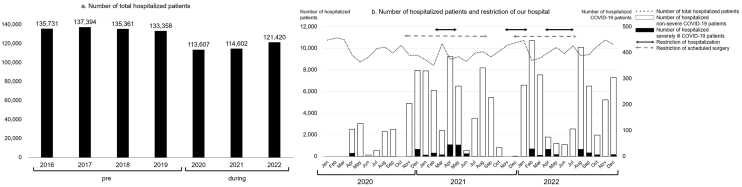
Changes in the number of inpatients. a. Annual number of total hospitalized patients in the pre- and during the pandemic periods. b. Monthly number of hospitalized patients during the pandemic (total and COVID-19 hospitalizations and the restrictions in hospitalizations and surgeries).

The annual number of hospitalized patients during the pandemic tended to decrease compared with that of the pre-pandemic period (Figure 3a). A survey on the number of patients with COVID-19 per month, severely ill patients with COVID-19, and patients without COVID-19 during the pandemic showed that as the number of patients with COVID-19 increased, restrictions on medical treatment led to fewer scheduled hospitalizations and surgeries (Figure 3b).

**Figure 4.**
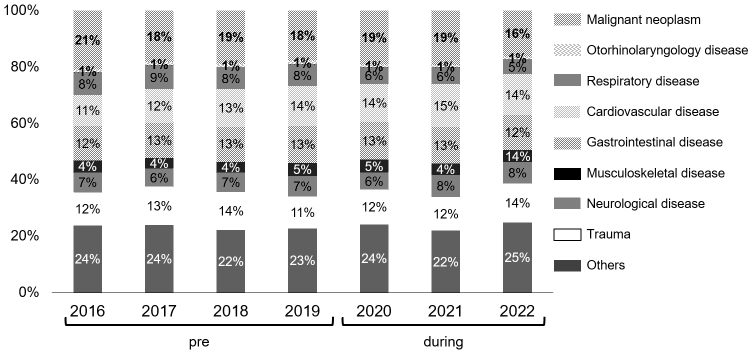
Disease composition of hospitalized patients in the pre- and during the pandemic periods. Diseases of patients treated in the hospital are represented with percentages. Note that the disease composition ratio did not show changes between the pre- and during the pandemic periods.

The total number of patients hospitalized for a condition other than COVID-19 during the pandemic tended to decrease overall compared with that of the pre-pandemic period (Figure 3a). However, the disease composition ratio did not clearly change (Figure 4).

### Influence of the pandemic on doctors working at Hyogo prefectural Awaji medical center

**Figure 5.**
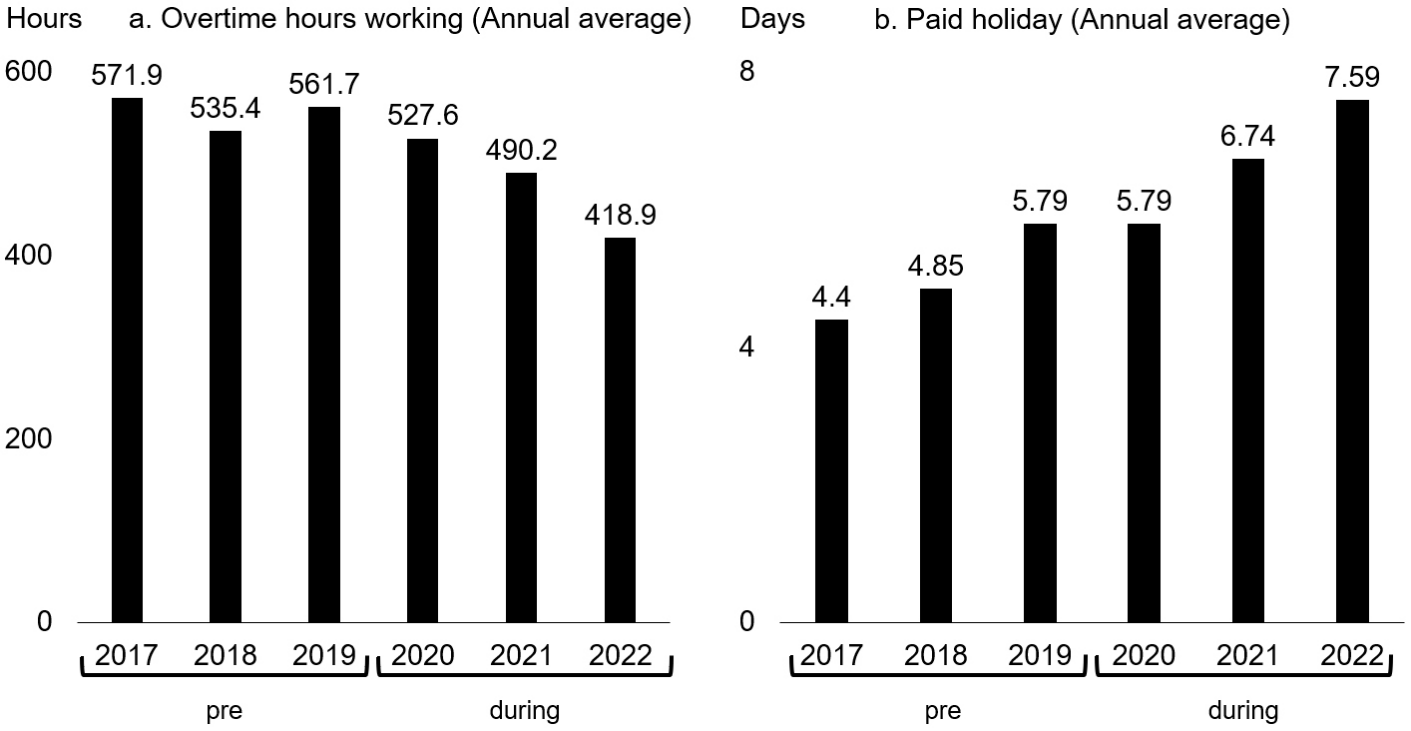
Changes in the work style of full-time doctors. a. overtime hours (annual average) b. paid holidays (annual average)

Figure 5 shows the annual overtime hours and days of paid leave taken by doctors working full time at our hospital in the pre- and during the pandemic periods. Doctors tended to work less overtime during the pandemic compared with the pre-pandemic period (Figure 5a). Conversely, the number of paid holidays taken by doctors tended to increase during the pandemic compared with the pre-pandemic period (Figure 5b).

We surveyed a total of 118 doctors working full time at our hospital. We received responses from 103 (80 men and 23 women) with 14.3 years of mean medical experience (median: 13 years), for a response rate of 87.2%. Among the 103 responses, items without a response were excluded. The contents and results of the survey are summarized in Table 1. Most of the respondents answered that there was no change in their working hours and sleep time during the pandemic. However, 26% of the doctors felt that their working hours had decreased, and 5% felt that their sleep time had increased. Additionally, 72% of the doctors responded that they had postponed a planned hospitalization, and 41% responded that their patients had consulted them about COVID-19. The contents of the consultations were about the timing of vaccinations and what to do if a family member was in close contact with a patient with COVID-19.

**Table 1.** Questionnaire Results of Full-Time Doctors Working at the Hyogo Prefectural Awaji Medical Center.

Questionnaire content	n	Answer
Change in working hours	103	Increased (7%)/No change (67%)/Decreased (26%)
Change in sleep time	102	Increased (5%)/No change (92%)/Decreased (3%)
Patient hospitalization postponed	100	Postponed (72%)/Not postponed (28%)
Consultation about COVID-19 from a patient	103	Yes (41%)/No (59%)

## Discussion

In this study, we conducted a survey of the epidemiological characteristics of COVID-19 on doctors working full time at Hyogo Prefectural Awaji Medical Center. The COVID-19 infection rate of Awaji Island was lower than those of Hyogo Prefecture and Japan as a whole, although the peaks occurred simultaneously. The number of outpatients and inpatients decreased during the pandemic because of restrictions on surgeries and hospitalizations, with no changes in the disease composition ratio. Regarding the doctors, overtime hours decreased, and the number of days of paid holiday taken increased.

The decrease in traffic volume on the Akashi Kaikyo Bridge and Onaruto Bridge due to social restrictions resulted in a decreased influx to Awaji Island during the pandemic. Furthermore, the limited transportation within Awaji Island might partially explain why the spread of infection decreased. Older adults aged ≥65 years comprise 38% of the total population of Awaji Island, which is 10% higher than that of the total population of Japan ^(5)^. The aging of the population is known to be associated with lower social activity levels and fewer opportunities for close contact with others. During the COVID-19 pandemic, people on Awaji Island did not seem to move around much, which could also help explain why the number of people on the island infected with COVID-19 remained relatively low. It was reported that older people living in rural areas tended to refrain from going out ^(10)^. Conversely, the fact that the COVID-19 infection rate on Awaji Island was the highest in August 2022 when compared with those of Hyogo Prefecture and Japan as a whole may be due to the increase in traffic on the Akashi Kaikyo Bridge and Onaruto Bridge.

As the COVID-19 infection rate increased on Awaji Island, both the number of ordinary and emergency outpatients at our hospital decreased (Figure 2). The reasons for this are thought to be the limited means of transportation on Awaji Island and the older adults refraining from receiving consultations, despite the fact that the COVID-19 countermeasures did not involve restrictions on outpatient care at our hospital. In Japan, as in other countries, the number of hospital beds was severely limited during the pandemic, and consultations were restricted. In response to these restrictions, some patients refrained from receiving medical examinations. Because the government issued a directive to citizens to “restrict nonessential outings,” many elderly people refrained from visiting our hospital. Patients’ vague anxiety at the time when little was known about COVID-19 may also be a factor ^(11)^. Bargon et al. ^(12)^ claimed that patients with cancer may become socially isolated as a result of refraining from seeing a doctor. Previous studies have reported that online consultations and home visits play a major role in understanding a patient’s physical and mental conditions ^(13), (14)^. However, online medical treatment systems have not been established at our hospital. In the beginning of the pandemic, although we only had a small number of patients, we sometimes took measures such as issuing prescriptions over the phone and having patients pick them up at a dedicated window, or making reservations for the next visit over the phone. These measures were likely meant to prevent the spread of infection or the exclusion of patients from medical care. Furthermore, a decrease in the number of opportunities to go out leads to an increased risk of older adults becoming completely withdrawn at home ^(15)^. Even in a pandemic situation, a system that allows patients with a disease other than COVID-19 to receive appropriate medical care is needed. In addition to triage at medical sites, various methods such as home visits, online medical consultations, and drive-through polymerase chain reaction tests were used around the world ^(16)^. The triage method is considered to be regulated by the number of patients with COVID-19 in the area and the availability of medical resources ^(16)^. However, it is important to consider that patients with conditions unrelated to COVID-19 may be at a disadvantage by imposing practice restrictions or closing ICUs for patients with COVID-19. According to Afschin ^(17)^, in the early stages of the COVID-19 pandemic, the admission of patients with COVID-19 to the ICU resulted in a loss of quality-adjusted life years due to missed treatment. Such an analysis is valid because the vaccine, treatment, and preventive methods for COVID-19 have been clarified to some extent. However, the possibility of future pandemics caused by novel pathogens other than COVID-19 cannot be ignored, so it is necessary to learn lessons from the recent COVID-19 pandemic and establish a medical system that does not require regular medical care as much as possible ^(18)^.

The reasons that the number of inpatients decreased (Figure 3a) were thought to be the decrease in outpatient visits and the restrictions on medical treatment caused by the COVID-19 countermeasure meetings. The COVID-19-dedicated ward was converted from a conventional tuberculosis ward. Further, after the dedicated wards became unable to accommodate all patients with COVID-19, general wards on the same floor as the dedicated wards were converted for patients with COVID-19. These conversions reduced the number of hospital beds available for patients with conditions or diseases other than COVID-19. Since the ICU was temporarily used for the most severely ill patients with COVID-19 (Figure 3b), it became crowded, which made it difficult to accept other patients with other diseases. However, without any restrictions on medical practice, the rate of hospital-acquired COVID-19 infections and thereby the infection rate on Awaji Island would have increased. There is also a report that Japan has been able to maintain one of the lowest infection rates in the world by adopting a system that determines acceptance based on available isolation beds for patients with COVID-19 ^(19)^. As a result of each department’s compliance with the consultation restrictions, no remarkable changes in disease composition were seen in the pre-pandemic period or during the pandemic (Figure 4).

At the COVID-19 countermeasures meeting, not only the restrictions on medical treatment, but also recommendations for medical departments to reduce the workload of doctors and for doctors to actively take paid holidays and go home on time, were discussed. Departments treating patients with COVID-19 experienced increased workloads, whereas others experienced the opposite. Specifically, in the surgical department, a reduction in the number of scheduled surgeries due to the restrictions resulted in a decreased workload. This might have affected changes in overtime hours and paid holidays taken in the pre- and during the pandemic periods. We did not find any previous studies that focused on the workload of full-time doctors due to medical treatment restrictions associated with COVID-19. However, there have been reports that the pandemic has led to more working hours and less vacation time for healthcare workers ^(20), (21), (22)^. In addition, many studies have reported that the pandemic led to more health problems among healthcare workers ^(23), (24), (25), (26), (27), (28)^. However, such signs were not often seen in our hospital, which seems to indicate that many full-time doctors properly restricted their practice when necessary and spent their free time meaningfully. Even without the COVID-19 pandemic, extended working hours can be a risk of burnout syndrome, so it is considered important to maintain a work-life balance ^(29)^. Additionally, sleep duration and work satisfaction are associated with burnout syndrome and psychological stress ^(30)^. Therefore, healthcare workers should be vigilant in protecting both their mental and physical health. A survey conducted in the UK found that healthcare workers had a satisfying clinical experience, even during the COVID-19 pandemic ^(31)^. However, in the present study, the results of the survey conducted on doctors working full time at our hospital indicated that they were not necessarily adversely affected (Table 1), their overtime hours decreased, and the number of days of paid holiday that they took have increased (Figure 5). The reason the COVID-19 pandemic did not adversely affect the doctors in our hospital could be their well-balanced work management skills. Together with the abovementioned restrictions on medical practice, it is important to consider ways to improve the satisfaction of healthcare workers and how to manage their working hours.

This study has some limitations. First, it was conducted in a single center on Awaji Island. Multicenter studies with hospitals that have different geographical conditions from our hospital may be needed to evaluate the characteristics of the COVID-19 pandemic by region. Second, we were not able to assess in detail how COVID-19-associated practice restrictions affected the care of patients with conditions or diseases other than COVID-19. Among these patients, those who were clearly hesitant to visit our hospital were contacted by their attending physician by telephone to issue prescriptions and make appointments for the next examination. These were incidents at the site level and could not be converted into analyzable data. Although our hospital was able to avoid a medical collapse due to the restrictions on medical care, we should be aware that behind the scenes, there may have been patients who fell victim to the restrictions on medical care. Third, the Medical Affairs Department refused to disclose individual data on overtime hours and the number of paid holidays from the viewpoint of personal information protection, which made it difficult to assess in detail the workload of each department due to the COVID-19 pandemic. Fourth, a survey on workers other than full-time doctors, who were not included in this study, should be conducted in the future. Fifth, data for the population influx from sea routes into Awaji Island were not obtained in the population survey. Sixth, it should be noted that the responses to the questionnaire conducted on doctors working full time at our hospital may have been affected by memory and subjective biases. Further studies are needed to prepare for future pandemics.

## Article Information

### Conflicts of Interest

None

### Acknowledgement

The authors gratefully acknowledge the help of their technical assistants for our survey at the Medical Affairs Department and General Affairs Division, and the doctors at Hyogo Prefectural Awaji Medical Center who responded to the questionnaires.

### Author Contributions

Conceptualization, Y.O., Y.S., M.S., H.N., and K.T.; Methodology, Y.O., N.O., M.S., H.N., and T.K.; Formal Analysis, Y.O., N.O., and M.S.; Investigation, Y.O. and N.O.; Data Curation, Y.O., N.O., M.S., and K.T.; Writing and Preparation of the Original Draft, Y.O., N.O., and K.T.; Writing Review and Editing, Y.O., N.O., Y.S., M.S., H.N., and K.T.; Visualization, Y.O. and N.O.; Supervision, K.T.; Project Administration, N.O.

### Approval by Institutional Review Board (IRB)

This study was approved by the Hyogo Prefectural Awaji Medical Center Ethics Committee (Approval No.: 22-1). This study was conducted in accordance with the principles of the Declaration of Helsinki.

## References

[1] World Health Organization. Pneumonia of unknown cause- China [Internet]. 2022 [cited 2023 Aug 22]. Available from: https://www.who.int/emergencies/disease-outbreak-news/item/2020-DON229.

[2] Rothan HA, Byrareddy SN. Epidemiology and pathogenesis of coronavirus disease (COVID-19). Res Microbiol. 2020;J4(2):675-87. 10.1016/j.jaut.2020.102433 PMC7127067 32113704

[3] Ministry of Health Labour and Welfare. COVID-19 infection medical guide. Version 8.1. 2022.

[4] Marinaccio A, Guerra R, Iavicoli S. Work a key determinant in COVID-19 risk. Lancet Glob Health. 2020;8(11):e1368. 32986980 10.1016/S2214-109X(20)30411-3 PMC7518834

[5] Hyogo Prefecture official HP elderly person health welfare-related document (aging rate) [Internet]. [cited 2023 Aug 22]. Available from: https://web.pref.hyogo.lg.jp/kf02/hw07_000000012.html.

[6] Hyogo Prefecture medical institution information system [Internet]. [cited 2023 Aug 22]. Available from: https://web.qq.pref.hyogo.lg.jp/hyogo/ap/qq/sho/pwmedregsr01_002.aspx.

[7] Ministry of Health Labour and Welfare learn from the data-Information about the new [Internet]. [cited 2023 Aug 22]. Available from: https://covid19.mhlw.go.jp/.

[8] Hyogo Prefecture official HP the outbreak situation of new coronavirus infectious disease [Internet]. [cited 2023 Aug 22]. Available from: https://web.pref.hyogo.lg.jp/kk03/corona_hasseijyokyo.html.

[9] Honshu-Shikoku bridge expressway company limited [Internet]. [cited 2023 Aug 22]. Available from: https://www.jb-honshi.co.jp/corp_index/company/data/traffic-result.html.

[10] Shimokihara S, Maruta M, Akasaki Y, et al. Association between frequency of going out and psychological condition among community-dwelling older adults after the COVID-19 pandemic in Japan. Healthcare (Basel). 2022;10(3):1-11. 10.3390/healthcare10030439 PMC8954262 35326917

[11] Ohta R, Ryu Y, Sano C. The uncertainty of Covid-19 inducing social fear and pressure on the continuity of rural, community-based medical education: A thematic analysis.Healthcare (Basel). 2021;9(2). 10.3390/healthcare9020223 PMC7922333 33671392

[12] Bargon CA, Batenburg MCT, van Stam LE, et al. Impact of the COVID-19 pandemic on patient-reported outcomes of breast cancer patients and survivors. JNCI Cancer Spectr. 2021;5(1):pkaa104. 33437925 10.1093/jncics/pkaa104 PMC7665619

[13] Wahezi SE, Kohan LR, Spektor B, et al. Telemedicine and current clinical practice trends in the COVID-19 pandemic. Best Pract Res Clin Anaesthesiol. 2021;35(3):307-19. 34511221 10.1016/j.bpa.2020.11.005 PMC7667401

[14] Wehrle CJ, Lee SW, Devarakonda AK, et al. Patient and physician attitudes toward telemedicine in cancer clinics following the COVID-19 pandemic. JCO Clin Cancer Inform. 2021;5:394-400. 33822651 10.1200/CCI.20.00183

[15] Mizutani M, Nishide R, Tanimura S, et al. Protective and high-risk social activities associated with homebound status among older adults in rural Japan. Prev Med Rep. 2022;30(November):102037. 36531108 10.1016/j.pmedr.2022.102037 PMC9747622

[16] Roque Mazoni S, Andrade J, da Silva Antonio P, et al. Triage strategies for COVID-19 cases: A scope review. Inquiry. 2022;59:469580221095824. 35549576 10.1177/00469580221095824 PMC9109280

[17] Gandjour A. COVID-19 and the forgone health benefits of elective operations. BMC Health Serv Res. 2022;22(1):1545. 36528629 10.1186/s12913-022-08956-6 PMC9759364

[18] Aron JA, Bulteel AJB, Clayman KA, et al. Strategies for responding to the COVID-19 pandemic in a rural health system in New York state. Healthcare. 2021;9(2):100508. 33711564 10.1016/j.hjdsi.2020.100508 PMC8055183

[19] Inoue H. Japanese strategy to COVID-19: How does it work? Glob Health Med. 2020;2(2):131-2. 33330791 10.35772/ghm.2020.01043 PMC7731332

[20] He Y, Yatsuya H, Chiang C, et al. The association of work-related stress according to the demand-control model with aggravation of pre-existing disease during the first state of Covid-19 emergency in japan. J Epidemiol. 2021;31(12):642-7. 34544998 10.2188/jea.JE20210146 PMC8593583

[21] Morawa E, Schug C, Geiser F, et al. Psychosocial burden and working conditions during the COVID-19 pandemic in Germany: The VOICE survey among 3678 health care workers in hospitals. J Psychosom Res. 2021;144:110415. 33743398 10.1016/j.jpsychores.2021.110415 PMC7944879

[22] Paiva T, Reis C, Feliciano A, et al. Sleep and awakening quality during COVID-19 confinement: Complexity and relevance for health and behavior. Int J Environ Res Public Health. 2021;18(7). 10.3390/ijerph18073506 PMC8037491 33800607

[23] Ing EB, Xu QA, Salimi A, et al. Physician deaths from corona virus (COVID-19) disease. Occup Med (Lond). 2020;70(5):370-4. 32409839 10.1093/occmed/kqaa088 PMC7239175

[24] Lasalvia A, Amaddeo F, Porru S, et al. Levels of burn-out among healthcare workers during the COVID-19 pandemic and their associated factors: A cross-sectional study in a tertiary hospital of a highly burdened area of north-east Italy. BMJ Open. 2021;11(1):e045127. 10.1136/bmjopen-2020-045127 PMC7813385 33455940

[25] Chatzittofis A, Karanikola M, Michailidou K, et al. Impact of the COVID-19 pandemic on the mental health of healthcare workers. Int J Environ Res Public Health. 2021;18(4):1-8. 10.3390/ijerph18041435 PMC7913751 33546513

[26] Sasaki N, Kuroda R, Tsuno K, et al. Deterioration in mental health under repeated COVID-19 outbreaks greatest in the less educated: A cohort study of Japanese employees. J Epidemiol. 2021;31(1):93-6. 33162424 10.2188/jea.JE20200499 PMC7738638

[27] Dimitriu MCT, Pantea-Stoian A, Smaranda AC, et al. Burnout syndrome in Romanian medical residents in time of the COVID-19 pandemic. Med Hypotheses. 2020;144:109972. 32531540 10.1016/j.mehy.2020.109972 PMC7276114

[28] Hummel S, Oetjen N, Du J, et al. Mental health among medical professionals during the COVID-19 pandemic in eight European countries: Cross-sectional survey study. J Med Internet Res. 2021;23(1):e24983. 33411670 10.2196/24983 PMC7817254

[29] Ishikawa M. Relationships between overwork, burnout and suicidal ideation among resident physicians in hospitals in Japan with medical residency programmes: A nationwide questionnaire-based survey. BMJ Open. 2022;12(3):e056283. 10.1136/bmjopen-2021-056283 PMC8915267 35273058

[30] Coelho J, Taillard J, Bernard A, et al. Emotional exhaustion, a proxy for burnout, is associated with sleep health in French healthcare workers without anxiety or depressive symptoms: A cross-sectional study. J Clin Med. 2023;12(5):1-12. 10.3390/jcm12051895 PMC10004252 36902682

[31] Bennett P, Noble S, Johnston S, et al. COVID-19 confessions: A qualitative exploration of healthcare workers experiences of working with COVID-19. BMJ Open. 2020;10(12):e043949. 10.1136/bmjopen-2020-043949 PMC7745452 33328264

